# The burden of aortic aneurysm in China from 1990 to 2019: findings from the Global Burden of Disease Study 2019

**DOI:** 10.1186/s12889-022-13221-w

**Published:** 2022-04-18

**Authors:** Xinran Hou, Fan Zhang, Zhi Ye, Qian Xu, Lingjin Huang, Qulian Guo, Wei Liu, Lijun Wang, Maigeng Zhou, Peng Yin, Maoen Zhu

**Affiliations:** 1 Department of Anesthesiology, Xiangya Hospital, Central South University, Changsha, Hunan P.R. China 410008; 2 Department of Cardiovascular Surgery, Xiangya Hospital, Central South University, Changsha, Hunan P.R. China 410008; 3 National Center for Chronic and Noncommunicable Disease Control and Prevention, Chinese Center for Disease Control and Prevention, Beijing, 100050 China

**Keywords:** Aortic aneurysm, Global Burden of Disease Study, Mortality, China

## Abstract

**Background:**

Aortic aneurysm (AA) is a global public health concern. However, little is known about the disease burden of AA in China.

**Methods:**

Following the general analytic strategy used in the Global Burden of Disease Study (GBD) 2019, we analyzed the mortality and years of life lost (YLLs) due to AA, stratified by sex, age, and province-level region in China from 1990 to 2019. The temporal trend of AA burden in China was analyzed and the main attributable risk factors for AA in China were also explored.

**Results:**

In China, the total AA deaths were 17,038 (95% UI: 14,392-19,980) in 2019, an increase of 136.1% compared with that in 1990, with an age-standardized death rate (ASDR) of 0.93 (95% UI: 0.79-1.08) per 100,000 person-years in 2019, a decrease of 6.8%. Meanwhile AA caused 378,578 (95% UI: 315,980-450,479) YLLs in 2019, an increase of 102.6% compared with that in 1990, with a crude YLL rate of 26.6 (95% UI: 22.2-31.7) per 100,000 person-years, an increase of 68.6%. The AA mortality and YLLs were higher in males than in females. AA caused most YLLs in the 65- to 75-year-old age group. The AA mortality and YLLs varied significantly among provinces in China, and the change in ASDR showed a negative correlation with the sociodemographic index of different provinces, namely, more decline of ASDR in developed provinces. High systolic blood pressure was shown to be the most significant attributable risk factor for AA burden in both males and females, and smoking was another major attributable risk factor, especially in males.

**Conclusions:**

The disease burden of AA increased significantly from 1990 to 2019 in China, with higher mortality and YLLs in males, senior populations, and among residents of most western provinces in China. High systolic blood pressure and smoking were two major attributable risk factors for AA mortality in China.

**Supplementary Information:**

The online version contains supplementary material available at 10.1186/s12889-022-13221-w.

## Background

Aortic aneurysm (AA) is characterized by permanent full-thickness dilation of the aortic wall, greater than 50% in diameter of normal size, and it can be generally classified into thoracic aortic aneurysm (TAA) and abdominal aortic aneurysm (AAA) according to the involved segments [1]. Although most AAs are asymptomatic at the time of diagnosis, the incidence of complications increases as aneurysms expand gradually [2–4]. The complications of AA, namely, dissection and rupture, are usually catastrophic, with mortality of 48%-56%, even when provided with instant emergent medical interventions [5]. For elective AAA repair, the 28-day mortality rate was reported to be 3.3%-27.1% in men and 3.8%-54.3% in women in a Dutch population [6]; in China, the overall 30-day mortality of infrarenal AAA repair was approximately 8.8% (28/316) in a single vascular center [7]. In an investigation of the general American population, the AAA-related mortality rate was 2.2 deaths per 100,000 in 2016 [8]. China has the largest and rapidly aging population, with a total population at the 2020 population census of 1.4 billion and a proportion of elder than 60 years old of 18.7% according to the lately released data of the seventh national population census [9], but epidemiologic surveys about the mortality or disease burden analysis of AA are inadequate.

The Global Burden of Diseases, Injuries and Risk Factors Study (GBD) provides a comprehensive assessment of the mortality and disability resulting from diseases, injuries, and risk factors based on published, publicly available, and contributed data worldwide [10]. GBD 2019 provides the most up-to-date assessment of the burden of 369 diseases and injuries for 204 countries and territories from 1990 to 2019 [10]. AA is one of the major cardiovascular diseases with an increased number of years of life lost (YLLs) and deaths globally [11]. Previously, a few studies analyzed the mortality trend of AA at the global and national levels using data derived from GBD 2010 [12] and GBD 2017 [13, 14], but studies on the AA burden in China are scarce.

Thus, in the present study, we assessed the burden of AA in China, measured by mortality and YLLs from 1990 to 2019, aiming to provide a reference for policy design and health resource allocation.

## Methods

### Overview

All the data analyzed in this study were derived from GBD 2019 [10], and the general methods, including details about the data, approaches to enhancing data quality and comparability, and statistical modeling and metrics for GBD 2019, have been reported previously [10, 15, 16]. A brief overview specific to the AA burden is presented here.

### Definitions

In the GBD 2019 hierarchy regarding the cause list of diseases and injuries, AA is at Level 3 with no further subdivision under cardiovascular diseases (Level 2) and noncommunicable diseases (Level 1). In the present study, International Classification of Diseases (ICD)-10th revision codes I71-I71.9 and ICD-9th revision codes 441-441.9 were used to define GBD 2019 aortic aneurysm causes under code B.2.9.

### Mortality and YLLs estimates

The detailed approach to estimating mortality in GBD studies has been described previously [10, 17]. Briefly, all data sources were mapped to the GBD cause list of diseases and injuries, followed by the redistribution of garbage codes or ill-defined causes. Then, the GBD Cause of Death Ensemble model (CODEm) method was used to generate estimates by age, sex, location, year, and cause. The main data sources for all causes of death in China included the China disease surveillance points (DSP) system, vital registration collected by the Chinese Center for Disease Control and Prevention (CDC), and medical certification of causes of death for Macao and Hong Kong [10, 15, 17]. YLLs were estimated as the number of deaths in each age group multiplied by the remaining life expectancy at the age of death from the reference life table. Since patients with AA were usually asymptomatic unless complications occurred, accompanied by a high fatality rate, years lived with disability (YLD) was not applicable to the assessment of AA burden, explained previously elsewhere [12]. Thus, mortality and YLLs, instead of disability-adjusted life years (DALYs), were used to measure the AA burden in this paper.

The 95% uncertainty intervals (UIs) were also supplied for each quantity, calculated by taking the 25th- and 975th-ordered draws in 1000 samples from the posterior distribution of each quantity [10].

### Temporal trends of AA burden worldwide and in China

The Joinpoint software (version 4.9.0.0. March 2021; Statistical Research and Applications Branch, National Cancer Institute) was used to perform Joinpoint regression analysis [18] to assess the slope of trends in the age-standardized death rate (ASDR) of AA during 1990-2019. The annual percentage change (APC) and the average APC (AAPC), both with 95% confidence intervals (CIs), were calculated to describe the temporal trend. The temporal trend of the disease burden of AA in China in the next ten years was predicted by the Auto-Regressive Integrated Moving Average (ARIMA) model, which has been widely used to forecast the trend of epidemiologic data [19, 20]. It was performed on the open-source R program (version 4.0.2) with forecast (version 8.15) and tseries (version 0.10–49) packages and the function, auto.arima(), to determine the optimal value of p, d, and q in ARIMA(p, d, q) model.

### Spatial pattern of AA burden at the subnational level

In China, data from 33 province-level administrative units, including 31 mainland provinces, municipalities, and autonomous regions, together with Hong Kong and Macao Special Administrative Regions (SAR), collectively referred to as the provinces, were analyzed and mapped.

### Correlation analysis

The sociodemographic index (SDI) is a composited indicator, ranging from 0 to 1, calculated by the equally weighted geometric mean of lag-distributed income per capita, average years of education in the population older than 15 years old, and total fertility rate among women younger than 25 years old [15]. Thus, the SDI reflects the social development of provinces and is shown on the map (Supplementary Figure 1). Moreover, according to geography, climate, economy, and culture, these provinces were also roughly classified into four regions: East (*n* = 12), Central (*n* = 6), West (*n* = 12), and Northeast (*n* = 3) (Supplementary Figure 1).

To explore the potential relationship between the AA burden and the social development level of different provinces, associations between the ASDR of AA and the SDI were evaluated by Spearman’s rho coefficients. A P value less than 0.05 was considered statistically significant.

### Attributable risk factors for AA

The ASDRs attributed to different risk factors for AA were collected from the GBD 2019 risk factor study datasets. The general methods have been detailed by the GBD 2019 Risk Factor Collaborators [16]. Briefly, the comparative risk assessment conceptual framework was used, and risk-outcome pairs of convincing or probable evidence were included. The relative risk by cause for mortality was collected from randomized controlled trials, cohorts, pooled cohorts, and case-control studies by a meta-regression-Bayesian regularized trimmed (MR-BRT) model. After adjustment, the prevalence of exposure was estimated in DisMod-MR 2.1 using a spatiotemporal Gaussian process regression (ST-GPR) model. The population attributable fractions (PAFs) were estimated relative to the counterfactual scenario of the theoretical minimum risk exposure level (TMREL) [16].

### Data visualization

Data visualization was performed by GraphPad Prism (version 8.0; GraphPad Software, Inc., USA), ArcGIS (version 10.2; Environmental Systems Research Institute, Inc., USA), R program (version 4.0.2; R Foundation for Statistical Computing), and Adobe Illustrator CS6 (version 16.0.0; Adobe Systems Inc., USA).

## Results

### Overall findings about Deaths and YLLs caused by AA

As shown in Table 1, AA was responsible for 172,427 (95% UI: 157,357-182,899) deaths globally in 2019, an increase of 82.1% compared with that in 1990, with an ASDR of 2.21 (95% UI: 2-2.35) per 100,000 person-years in 2019, a decrease of 17.9%. Meanwhile, AA caused 3,322,343 (95% UI: 3,107,725-3,524,925) YLLs in 2019, an increase of 67% compared with that in 1990, with a crude YLL rate of 42.9 (95% UI: 40.2-45.6) per 100,000 person-years, an increase of 15.5%.

**Table 1:** Disease burden of AA in the world and in China, 1990-2019

	All-Age Deaths, Number (95% UI)		ASDR (95% UI)		YLLs, Number (95% UI)		YLLs, Rate (95% UI)	
	2019 (cases)	Change from 1990 (%)	2019 (per 100 000 person-years)	Change from 1990 (%)	2019 (person-years)	Change form 1990 (%)	2019 (per 100 000 person-years)	Change from 1990 (%)
World								
Male	108341 (100182-114716)	71.6	3.15 (2.89-3.35)	-24.7	2252449 (2099537-2400190)	61.2	58 (54.1-61.8)	11.9
Female	64086 (55525-69669)	103.1	1.46 (1.27-1.59)	-9.5	1069894 (969436-1158252)	80.7	27.7 (25.1-30)	24.5
Both	172427 (157357-182899)	82.1	2.21 (2-2.35)	-17.9	3322343 (3107725-3524925)	67	42.9 (40.2-45.6)	15.5
China								
Male	12089 (9620-14991)	146.9	1.48 (1.2-1.8)	0.2	279763 (220489-352085)	111.4	38.6 (30.4-48.6)	78
Female	4949 (4022-5967)	113.5	0.51 (0.41-0.61)	-19.9	98815 (78342-121355)	81.1	14.2 (11.2-17.4)	48.9
Both	17038 (14392-19980)	136.1	0.93 (0.79-1.08)	-6.8	378578 (315980-450479)	102.6	26.6 (22.2-31.7)	68.6

*ASDR,* age-standardized death rate; *YLLs*, years of life lost, *95% UI* 95% uncertainty intervals

By comparison, in China, AA was responsible for 17,038 (95% UI: 14,392-19,980) deaths in 2019, an increase of 136.1% compared with that in 1990, with an ASDR of 0.93 (95% UI: 0.79-1.08) per 100,000 person-years in 2019, a decrease of 6.8%. Meanwhile, AA caused 378,578 (95% UI: 315,980-450,479) YLLs in 2019, an increase of 102.6% compared with that in 1990, with a crude YLL rate of 26.6 (95% UI: 22.2-31.7) per 100,000 person-years, an increase of 68.6%.

### Temporal trends of AA burden among males and females from 1990 to 2019 and the forecasts for the next ten years in China

**Fig. 1:**
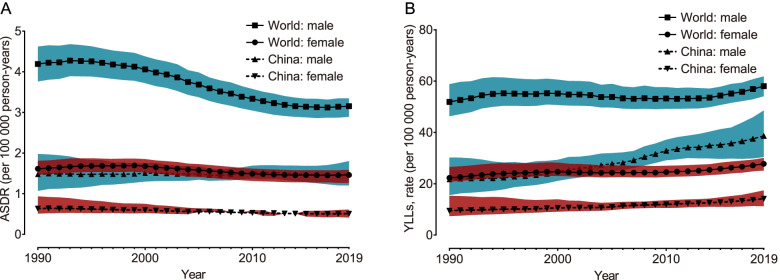
The temporal trend of ASDR and crude YLL rate caused by AA from 1990 to 2019 stratified by sex. **A** The temporal trend of ASDR due to AA in the world and in China; **B** The temporal trend of the YLL rate due to AA in the world and in China. ASDR, age-standardized death rate; YLLs, years of life lost

As shown by Joinpoint regression analysis (Supplementary Table 1), globally, the AAPC of ASDR due to AA was -0.7% (95% CI: -0.8% to -0.6%) with -1.0% (95% CI: -1.1% to -0.9%) in males and -0.3% (95% CI: -0.4% to -0.3%) in females and -0.3% (95% CI: -0.4% to -0.1%) in China with 0 (95% CI: -0.2% to -0.2%) in males and -0.8% (95% CI: -0.9% to -0.7%) in females. The absolute values of the ASDR and YLL rates in China were relatively low, but the disease burden of AA in China did not decrease obviously, particularly in males; meanwhile, the disease burden of AA was much higher in males than in females (Fig. 1).

**Fig. 2:**
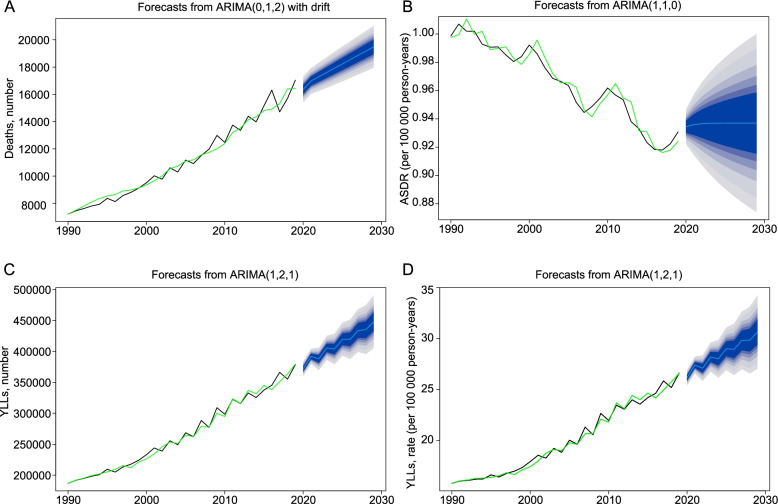
Forecasts of the disease burden of AA in the next ten years in terms of deaths (**A**), ASDR (**B**), YLLs (**C**), and crude rate of YLLs (D) by ARIMA model. The black lines represent estimates from GBD 2019; the green lines indicate fitted curves by the ARIMA model. The blue lines indicate forecasts for the next ten years by the ARIMA model, with shaded areas from inside to outside indicating confidence levels of 50%, 60%,70%, 80%, 90%, 95% respectively. ASDR, age-standardized death rate; YLLs, years of life lost; ARIMA, Auto-Regressive Integrated Moving Average

The disease burden of AA in China in the next ten years was predicted by the ARIMA model, which fitted well with GBD 2019 estimations from 1990 to 2019, and it could be expected that a continuous increase in the absolute number of deaths and YLLs and the crude YLL rate would be inevitable, while the ASDR would keep stable by 2029 (Fig. 2).

### Age-specific burden of AA in males and females from 1990 to 2019

As shown in Supplementary Table 2, AA caused more deaths in the elderly groups, especially those over 65, and peaked in those aged 70-79 in males and 75-84 in females in 2019. In recent decades, the proportion of the aged population in deaths caused by AA has expanded steadily (Supplementary Figure 2). In nearly all age groups, the absolute number of deaths due to AA surpassed that in 1990, moreover, the mortality rates in 2019 were also higher than those in 1990 in quite a few middle- and advanced-age groups for males (Supplementary Table 2).

**Fig. 3:**
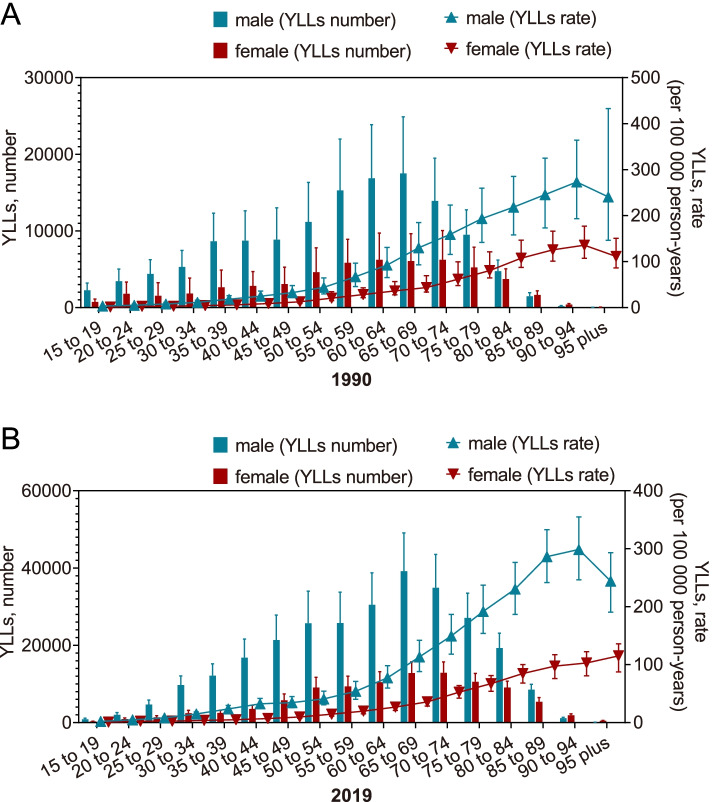
The comparison of the distribution of YLLs caused by AA in different age groups and the curve of the crude YLL rate due to AA corresponding to the age groups in 1990 (**A**) and in 2019 (**B**). YLLs, Years of life lost

AA caused most YLLs in both males and females aged 65-75, and the YLL rate increased with age similarly in both males and females except for the superaged groups already far beyond the average life expectancy. In most age groups, both male and female, the overall value of YLLs due to AA in 2019 exceeded that in 1990. The peak YLLs shifted from the 60-69 age group in 1990 to the 65-74 age group in 2019 for both males and females (Supplementary Table 2 and Fig. 3).

### Spatial pattern of AA burden and its changes from 1990 to 2019

**Fig. 4:**
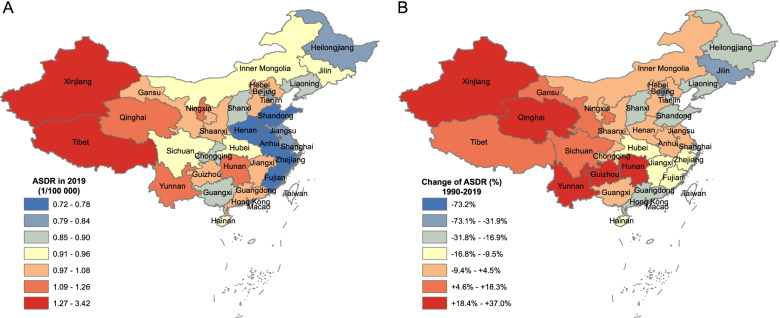
The geographic distribution of ASDR caused by AA at the province level in 2019 (**A**) and the percentage of change in ASDR compared with that in 1990 (**B**). ASDR, age-standardized death rate

There were noteworthy disparities in AA-caused deaths, YLLs, and changes of them across all provinces from 1990 to 2019. In 2019, the provinces with the highest ASDR were Macao (3.42, 95% UI: 2.41-4.67), Xinjiang (2.23, 95% UI: 1.77-2.71), Tibet (2.18, 95% UI: 1.83-2.6), Qinghai (1.26, 95% UI: 1.02-1.5), and Yunnan (1.24, 95% UI: 1.03-1.48), while the largest increases occurred in Xinjiang (37%), Qinghai (28%), Hunan (27.5%), Yunnan (22.7%), and Guizhou (22.2%), which were mainly located in northwestern and southwestern China. The rank of the crude YLL rate was similar to that of ASDR, with the top five being Macao (101.3, 95% UI: 70.8-141.9), Xinjiang (59.4, 95% UI: 44.9-74.1), Tibet (47.7, 95% UI: 38.2-59.2), Qinghai (31.6, 95% UI: 24.2-41.2), and Hunan (34.9, 95% UI: 27.4-43.8) (Supplementary Table 3 and Fig. 4).

### Correlation analysis of AA burden and SDI

**Fig. 5:**
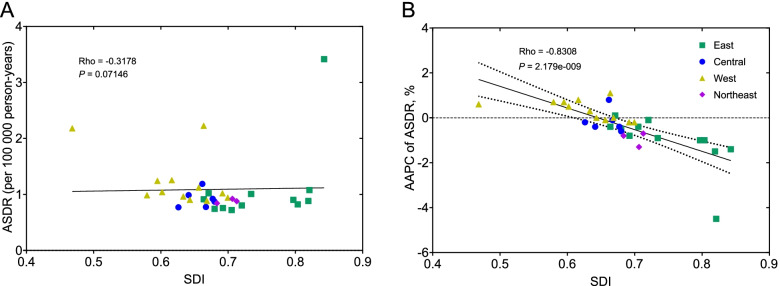
The correlation of ASDR caused by AA in 2019 (**A**) and the AAPC of ASDR during 1990-2019 (**B**) with the SDI of different provinces in China. ASDR, age-standardized death rate; AAPC, average annual percentage change; SDI, sociodemographic index

In cross-sectional observations, the ASDR of AA in 2019 showed no obvious correlation with the SDI. However, when focused on the dynamic change in ASDR from 1990 to 2019, a consistently negative correlation between the AAPC of ASDR and SDI was observed (Rho = -0.8308, *P* < 0.0001), demonstrating that the ASDR due to AA declined faster in provinces with higher SDI (Fig. 5).

### Attributable risk factors for AA burden from 1990 to 2019

**Fig. 6:**
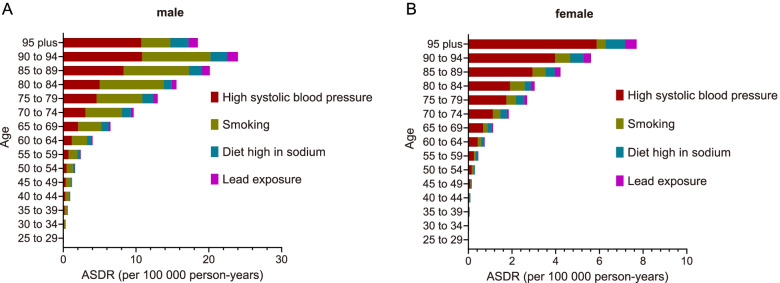
The attributed ASDR of risk factors for AA in 2019 in different age groups in males (**A**) and females (**B**). ASDR, age-standardized death rate

To further explore the composition of AA burden, attributable risk factors were analyzed, and high systolic blood pressure, smoking, diet high in sodium, and lead exposure in the risk factor hierarchy were screened out. High systolic blood pressure was the most significant attributable risk factor in both males and females, and the proportion increased with age (Fig. 6). Notably, smoking was another major attributable risk factor for AA burden, especially in males (Fig. 6), while a diet high in sodium and lead exposure constituted a relatively lower proportion. In addition, from 1990 to 2019, there was no significant decrease in attributable ASDR of AA for high systolic blood pressure or smoking, and there was even a slight uptrend for males (Supplementary Figure 3).

## Discussion

In the present study, a substantial increase in deaths and YLLs due to AA and a slight decrease in ASDR in 2019 were found in China compared with that in 1990. The AA burden was much higher in males and older age groups and populations in western and less-developed provinces. High systolic blood pressure and smoking were two leading attributable risk factors for AA.

From 1990 to 2019, the substantial increase in deaths and YLLs in China is mainly attributable to general population growth and aging. It was estimated that in China, the population increased from 1.1 billion in 1990 to 1.4 billion in 2019, and the life expectancy at birth increased from 68.1 years in 1990 to 77.6 years in 2019 [15]. The deaths due to AA in China comprised nearly 10% of that in the world, though the mortality rate was relatively low, which might be explained by racial differences [21] and the availability of screening and preventive health care [11]. Noticeably, the ASDR due to AA in China did not decline remarkably, like the global average, which might be a result of multiple factors, such as the large population base in China, the aging of the population, and advances in diagnosis.

In 2019, the mortality rate due to AA in males was almost triple that of females, in line with global data [11, 13], possibly due to risk factor exposure in men, such as smoking, hypertension, and hypercholesteremia [22]. It was reported that the sex differences in aortic aneurysm formation were influenced by sex hormones [23]. Additionally, it was also reported that women with thoracic or abdominal aortic aneurysms have a higher risk of acute aortic syndromes, higher perioperative mortality, and more major complications [24–26].

The mortality rate increased consistently with age in both males and females [12, 13]. Meanwhile, in line with the global trend [13], the percentage of elderly deaths from AA was growing (Supplementary Figure 2), and the population aged 60-80 sustained the highest YLLs due to AA in 2019, a shift from the most affected age group in 1990, aged 55-75, and this shift was mainly caused by the increased longevity of the population [15].

The ASDR varied among provinces. It was low in eastern coastal provinces with a few exceptions and relatively high in western and some central provinces. Provinces with both high ASDR and high growth rates, such as Xinjiang, Qinghai, Tibet, Yunnan, and Hunan, may need to be given additional attention in allocating the finite medical investments.

The discrepancy among regions, in which the ASDR due to AA ranged from 0.72 to 3.42 per 100,000 person-years in 2019 in different provinces, may be associated with sociodemographic development, but the correlation was weak and not statistically significant, which is partially explained by the differences in data sources, such as Hong Kong and Macao. The cross-sectional observation of ASDR in 2019 revealed higher rates in some high SDI regions, like Macao (SDI: 0.843) and Hong Kong (SDI: 0.821), possibly due to the higher completion rate for AA diagnosis before death. Meanwhile, the ASDR was high in some low SDI provinces, like Tibet (SDI: 0.468) and Xinjiang (SDI: 0.664), possibly due to the limited medical resources. Notably, the dynamic change in ASDR showed a negative correlation with the SDI, which means that the ASDR declined rapidly in those developed provinces. It was also observed on a global scale that the ASDR in high-income countries showed a more profound decline from 1990 to 2017 [14]. Thus, the burden of AA might be lighter in areas with sustained social development.

The reported risk factors for AA included male sex [27, 28], white race [28], smoking [27, 28], hypertension [27], high low-density lipoprotein [28], and hypercholesteremia [28–30]. For the Chinese population, it was reported that age, smoking habits, degree of hypertension, and poor control of hypertension were high-risk factors for the formation and progression of AAA [31]. In our present study, the most predominant attributable risk factors for AA burden were high systolic blood pressure and smoking; in addition, the AA-induced death rate attributed to these two risk factors was higher in males. In China, there is a high prevalence of hypertension, and low awareness, treatment, and control of it. According to surveys, 23.2% of the Chinese adult population (≥18 years of age) had hypertension and among them, only 46.9% were aware of their diagnosis; 40.7% were taking prescribed antihypertensive medications; 15.3% had controlled hypertension [32]. Similarly, for adults aged 35-75 years from all 31 provinces in mainland China, the prevalence of hypertension was 44.7%, with 44.7% awareness, 30.1% treatment, and 7.2% control [33]. Meanwhile, China has the world's most smokers and the standardized smoking prevalence was estimated to be 24.9%-26.0% in the overall population aged 15 years or older and 47.0%-48.4% in men [34]. For the temporal trend of attributable risk factors, the expected decline was not as pronounced as that in high SDI countries [13]. In addition, the death rate attributed to high systolic blood pressure and smoking increased with age. Therefore, emphasis on smoking prevention or cessation and blood pressure control is warranted to reduce the AA burden in China, especially in the male and elderly populations.

For the early detection of AA, ultrasound screening might be beneficial for specific populations at risk, with a 42%-48% risk reduction in aneurysm-related deaths in a sample of men (*n*=67,800) aged 65-74 years [35, 36], and the effect might be linked primarily to the initiation of pharmacological therapy [37]. For the growth rates of AA, it was estimated to be 0.28 cm/year in small AAA (diameters < 5.0 cm) and 0.75 cm/year in large AAA (diameters ≥ 5.0 cm) in Chinese population [38]. For the treatment of AA in patients with small (3.0-5.4 cm) and stable AAAs, surgical intervention showed no more benefits compared with surveillance [39, 40]; for patients with operative indications, endovascular aneurysm repair showed lower perioperative mortality and similar long-term overall survival compared with traditional open repair [41].

However, some limitations existed in this study. First, the diagnosis of AA may have not been finished when patients died, especially in some less-developed regions, which could lead to the underestimation of AA mortality. Second, the incidence and prevalence of AA were not estimated because of the paucity of epidemiologic data from China. Third, most of the data were derived from AAA, but TAA exhibited some distinctions from pathogenesis to prognosis, although less prevalent [2, 42]; therefore, specialized surveys on TAA in China are needed. Fourth, the analysis of the risk factors for AA was somewhat speculative, and the causality should be confirmed specifically.

## Conclusions

Aortic aneurysm is still one of the major fatal cardiovascular diseases, and the ASDR of AA in China demonstrated a relatively modest decline from 1990 to 2019. Males and elderly individuals showed higher mortality rates of AA in China. Most western provinces and some central provinces need additional medical resources. High systolic blood pressure and smoking were two major attributable risk factors for AA mortality and should be emphasized in AA-relevant public health policy formulations.

## Supplementary Information

**Additional file 1.** 

**Additional file 2.** 

**Additional file 3.** 

**Additional file 4.** 

**Additional file 5.** 

**Additional file 6.** 

## Data Availability

The datasets analyzed during the current study are available on the website of the Global Health Data Exchange (http://ghdx.healthdata.org).

## Abbreviations

AA: Aortic aneurysm
GBD: Global Burden of Disease, Injuries and Risk Factors Study
YLL: Years of life lost
ASDR: Age-standardized death rate
SDI: Sociodemographic Index
TAA: Thoracic aortic aneurysm
AAA: Abdominal aortic aneurysm
ICD: International Classification of Diseases
DALYs: Disability-adjusted life years
YLD: Years lived with disability
UI: Uncertainty intervals
APC: Annual percentage change
AAPC: Average annual percentage change
ARIMA: Auto-Regressive Integrated Moving Average
