# Supplementary figures and images for: The burden of aortic aneurysm in China from 1990 to 2019: findings from the Global Burden of Disease Study 2019
Source: BMC Public Health. 2022 Apr 18;22:782. doi: 10.1186/s12889-022-13221-w (PMC9016999; doi:10.1186/s12889-022-13221-w)

A

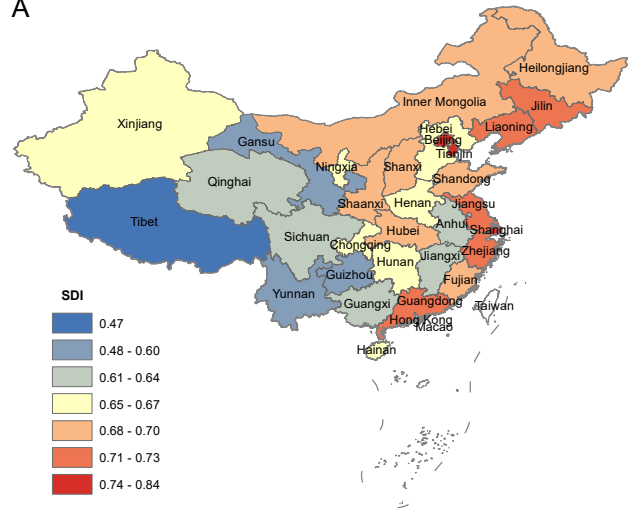

B

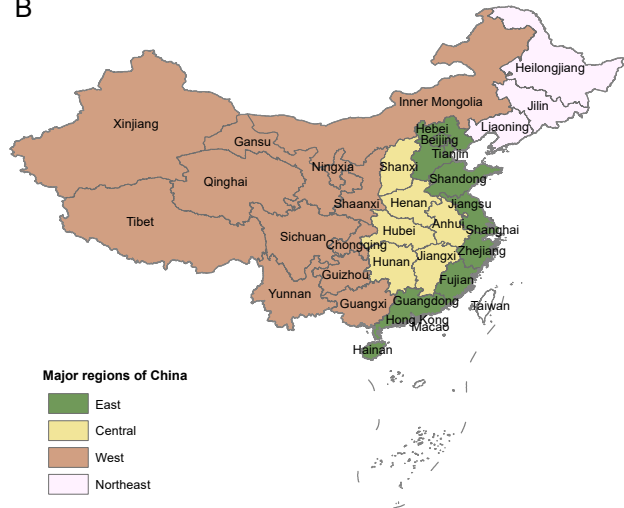

Supplement: Supplementary file 1 — Additional file 1. [file 12889_2022_13221_MOESM1_ESM.pdf]

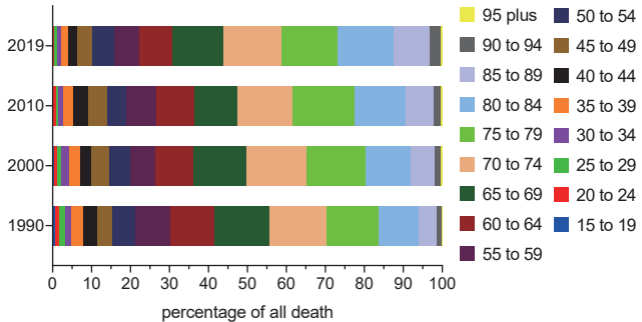

Supplement: Supplementary file 2 — Additional file 2. [file 12889_2022_13221_MOESM2_ESM.pdf]

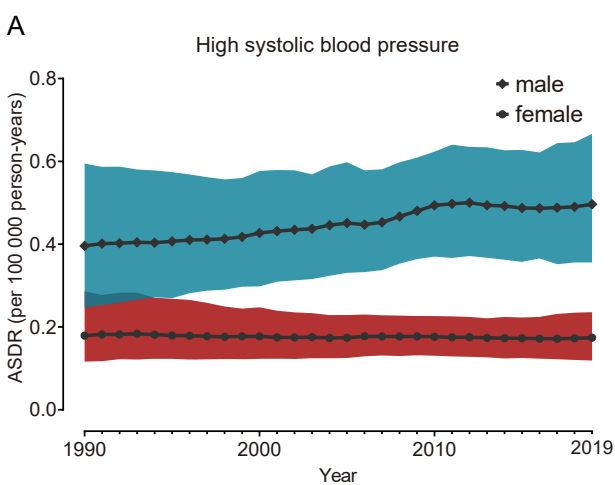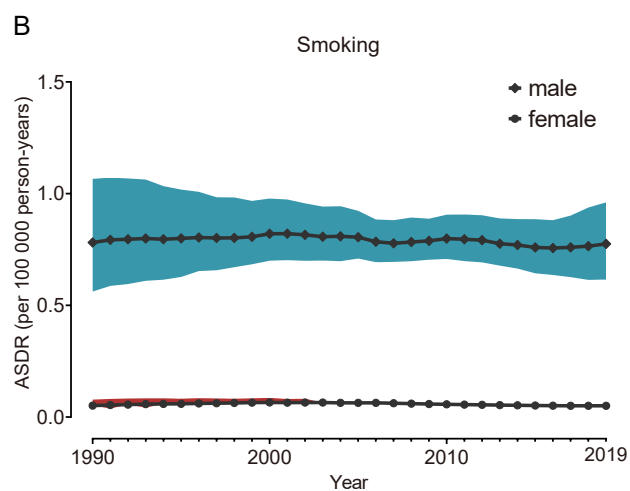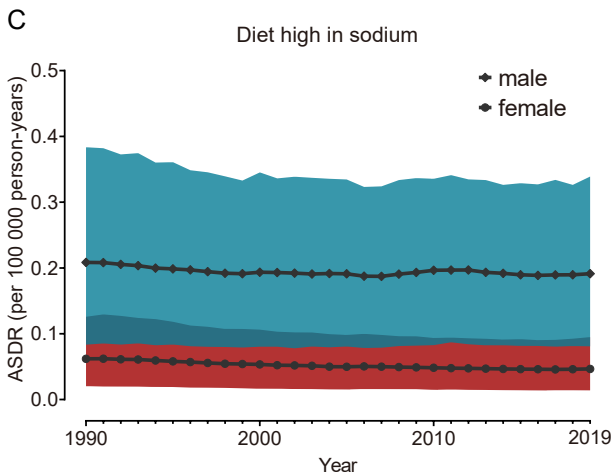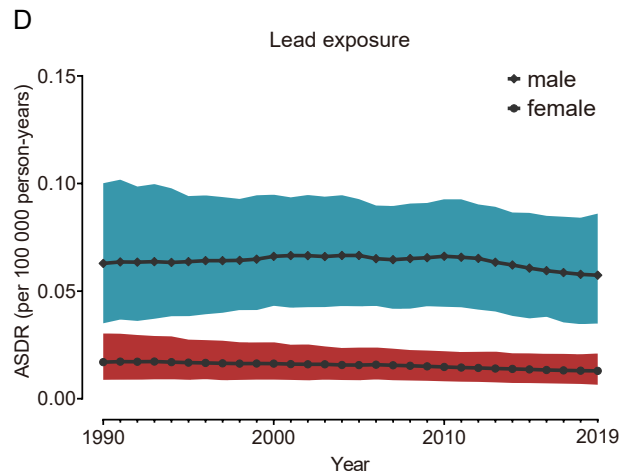

Supplement: Supplementary file 3 — Additional file 3. [file 12889_2022_13221_MOESM3_ESM.pdf]
